# Persistent Overexposure to *N*-Methyl-d-Aspartate (NMDA) Calcium-Dependently Downregulates Glutamine Synthetase, Aquaporin 4, and Kir4.1 Channel in Mouse Cortical Astrocytes

**DOI:** 10.1007/s12640-018-9958-3

**Published:** 2018-09-15

**Authors:** Katarzyna Skowrońska, Marta Obara-Michlewska, Anna Czarnecka, Katarzyna Dąbrowska, Magdalena Zielińska, Jan Albrecht

**Affiliations:** Department of Neurotoxicology, Mossakowski Medical Research Centre, Polish Academy of Sciences, Pawińskiego St. 5, 02-106 Warsaw, Poland

**Keywords:** Astrocytes, NMDA receptor, GluN1, Glutamine synthetase, Aquaporin 4, Kir4.1, Excitotoxicity

## Abstract

Astrocytes express *N*-methyl-d-aspartate (NMDA) receptor (NMDAR) but its functions in these cells are not well defined. This study shows that the sustained exposure (8–72 h) of mouse astrocytes to NMDA decreases the expression of the functional astroglia-specific proteins, glutamine synthetase (GS), and the water channel protein aquaporin-4 (AQP4) and also reduces GS activity. Similar to rat astrocytes (Obara-Michlewska et al. Neurochem Int 88:20–25, 2015), the exposure of mouse astrocytes to NMDA also decreased the expression of the inward rectifying potassium channel Kir4.1. NMDA failed to elicit the effects in those cells incubated in the absence of Ca^2+^ and in those in which the GluN1 subunit of the NMDAR was silenced with GluN1 siRNA. The downregulation of GS, AQP4, and Kir4.1 observed in vitro may reflect NMDAR-mediated alterations of astrocytic functions noted in central nervous system pathologies associated with increased glutamate (Glu) release and excitotoxic tissue damage.

## Introduction

The *N*-methyl-d-aspartate subtype of the ionotropic glutamate (Glu) receptor (NMDAR) plays a critical role in the central nervous system (CNS) function, and the excessive NMDAR activity contributes to the pathogenesis of CNS disorders related to the increased glutamatergic tone and excitotoxicity (recently reviewed by Gonzalez et al. 2015). For a long time, the interpretation of the role of NMDAR in the CNS has almost exclusively accounted for receptors that are located in neurons. Studies of the last two decades have provided the evidence for the expression of all major NMDAR subunits in astrocytes and the subunit composition of the receptor showing variation with tissue preparation and physiological context (Schipke et al. 2001; Krebs et al. 2003; Dzamba et al. 2013; Jimenez-Blasco et al. 2015). Astrocytic NMDAR is capable of generating specific currents and intra-astrocytic Ca^2+^ accumulation, which may occur either by a canonical, ionotropic (Lalo et al. 2006; Palygin et al. 2010), and/or metabotropic-like extracellular calcium-independent mechanism (Gerard and Hansson 2012; Montes de Oca Balderas and Aguilera 2015; Jimenez-Blasco et al. 2015). However, astrocytic NMDAR differs from its neuronal counterpart in the limited sensitivity to Mg^2+^ blockade and low Ca^2+^ permeability; the difference is most likely due to the relative overrepresentation of the triheteromeric (GluN1 + GluN2 + GluN3) composition of NMDAR in astrocytes (for reviews, see Verkhratsky and Kirchhoff 2007; Hogan-Cann and Anderson 2016).

The role of astrocytic NMDAR in the brain physiology and pathology has only begun to be understood. Under physiological conditions, astrocytic NMDAR plays a neuroprotective role by driving an intracellular mechanism eliciting increased supply of the antioxidant glutathione to neurons (Jimenez-Blasco et al. 2015) and appears to contribute to the synaptic plasticity regulation (Letellier et al. 2016). A few studies have analyzed responses of NMDAR in astrocytes to pathogenic stimuli. Astrocytic NMDAR increased secretion of the proinflammatory cytokines in the cells subjected to inflammatory stimulation (Gerard and Hansson 2012; Sühs et al. 2016), promoted the secretion of β-NGF in the amyloid β 1–40 treated hippocampus (Li et al. 2016), and contributed to the increased calcium fluxes in astrocytes subjected to the prolonged exposure to ammonia (Haack et al. 2014).

Our previous study demonstrated that a long-term exposure of cultured rat astrocytes to Glu and NMDA decreased the expression of Kir4.1, an astroglia-specific inward rectifying potassium channel (Obara-Michlewska et al. 2015). Basing on this finding, we hypothesized that the sustained overstimulation of astrocytic NMDAR may also induce alterations in the expression of other astrocytic proteins critical for maintenance of homeostatic functions of astrocytes in the CNS. In this study, therefore, we examined the effect of the exposure of cultured mouse astrocytes to NMDA on the expression of glutamine synthetase (GS) and aquaporin-4 (AQP4). GS controls the synthesis and inactivation of neurotransmitter amino acids Glu and GABA (Schousboe et al. 2013), while AQP4 regulates the water movement both into and out of astrocytes (Potokar et al. 2016) and the paravascular cerebrospinal fluid (CSF)/interstitial fluid (ISF) flow conducted by the glymphatic system (Iliff et al. 2012). To ascertain that the effects of NMDA are mediated by NMDAR, we also measured the responses in cultures with silenced GluN1, the subunit essential for the assembly of other NMDAR subunits and the formation of a functional NMDAR channel (Atlason et al. 2007). Preliminary analysis of the contribution of the ionotropic vs metabotropic mechanisms was performed by measuring the effects of NMDA in the presence and absence of calcium in the incubation medium. GS activity in NMDA-exposed astrocytes was measured to assess whether changes in its expression are translated to altered function.

## Materials and Methods

### Preparation of Astrocyte Cultures

**Fig. 1:**
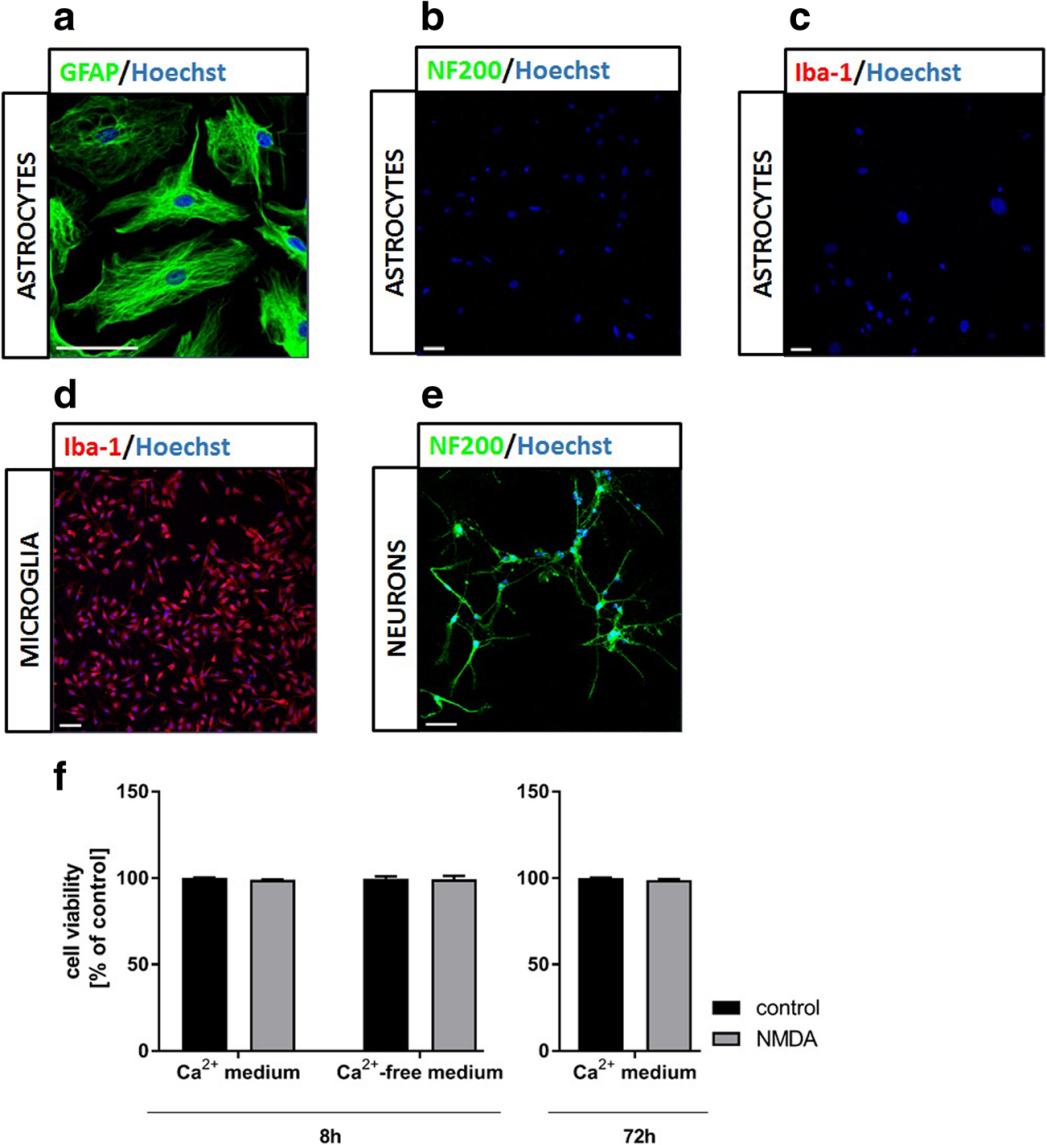
Immunocytochemical characterization of astrocytes and assessment of other cell contaminants in primary mouse astrocytic cultures (**a**–**c**), verification of antibodies against proteins of non-astrocytic mouse cortex cells (**d**, **e**) and MTT assay (**f**). Astrocytes were positively immunostained against the astrocytic filamentous protein GFAP (**a**), but negatively against the neuronal marker NF200 (**b**) and the microglial marker Iba-1 (**c**). Cultured mouse microglia (a kind gift of Prof. Bożena Kamińska, Nencki Institute of Experimental Biology, Warsaw) (**d**) and cultured mouse neurons (**e**) were stained with antibodies against Iba-1 and NF200, respectively. Blue Hoechst stain indicates the location of cell nuclei. Scale bars = 50 μm. **f** The effect of treatment with NMDA (100 μM) on the viability of cultured mouse cortical astrocytes (MTT assay). Data in (f) are mean (± SD) from four independent experiments and are expressed as % of control (standard medium)

**Fig. 2:**
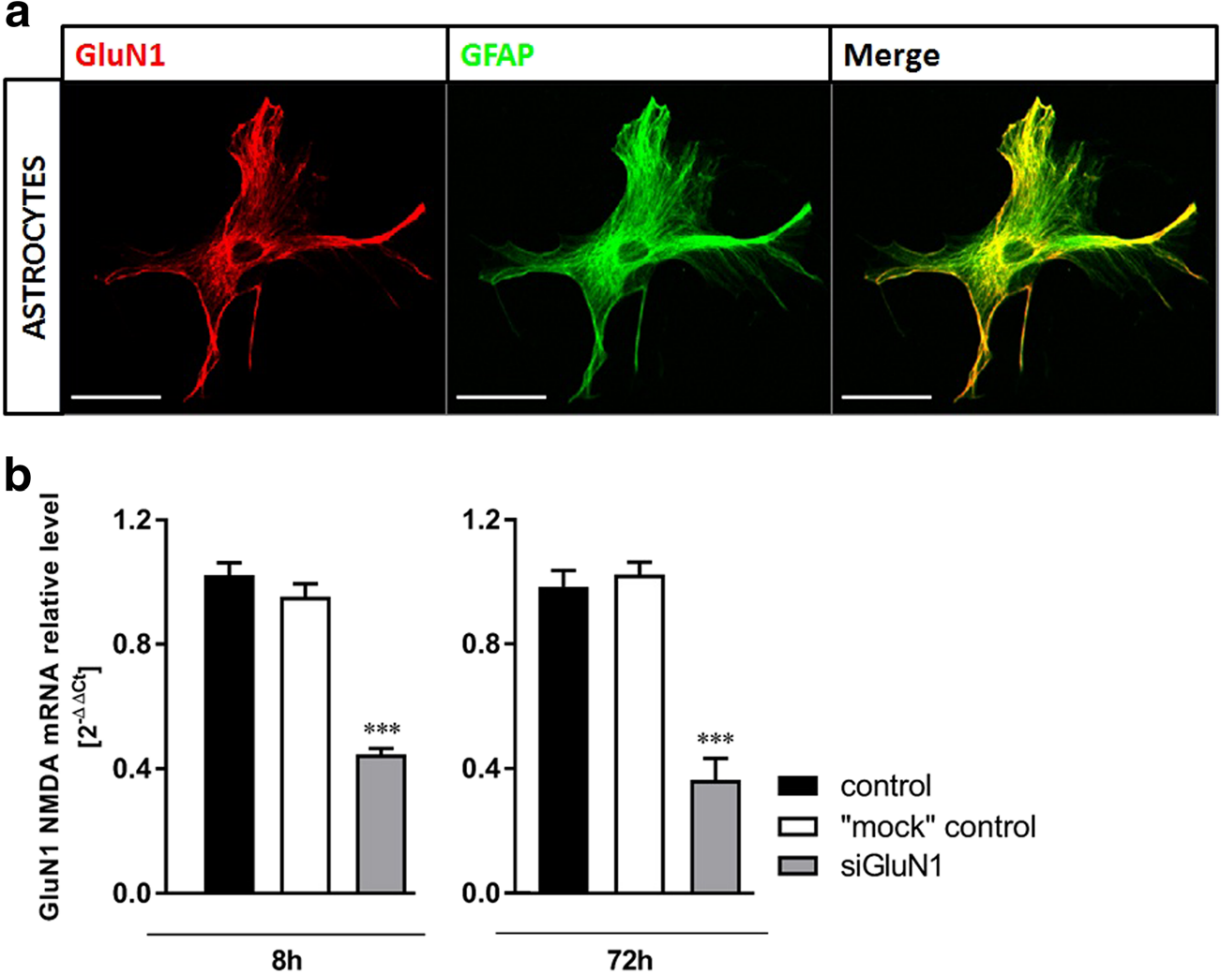
**a** The expression of GluN1 protein on the surface membrane of cultured astrocytes. Astrocytes were identified by immunostaining against the astrocytic filamentous protein GFAP. Image of GluN1 (red, left), GFAP (green, middle), and merged images (right) . Scale bars = 50 μm. **b** The effect of GluN1 subunit silencing with siRNA for 8 h and 72 h on the GluN1 mRNA content in cultured mouse cortical astrocytes. Data are mean (± SD) from three independent experiments. Significant difference vs. control, ***P < 0.001 (One-way ANOVA with the Dunnett post hoc test)

The mouse primary cerebral cortical astrocytes were cultured as described by Hertz et al. (1989). This procedure, rendering astrocytic preparation contaminated by less than 10% with other cell constituents (Hertz et al. 1989), was used in several studies characterizing metabolic events and drug-induced responses of astrocytes, as well as aspects of astrocytic-neuronal interactions (cf. Eloqayli et al. 2011; Gaul et al. 1992; Nicholson and Renton 2002; Pan et al. 2012; Waagepetersen et al. 2000; White et al. 2002). Briefly, 7-day-old C57BL6/J mice were rapidly decapitated and the brains were taken out. The cerebral cortex was aseptically separated from the remainder of the brain, and after the removal of the meninges, the tissue was sliced and reintroduced into Dulbecco’s modified Eagle’s medium (DMEM; Sigma-Aldrich, St. Louis, Missouri, USA) containing 20% fetal bovine serum (FBS; Bio-Tech, Warsaw, Poland), 100 units/mL of penicillin, and 100 μg/mL of streptomycin (Gibco, Thermo Fisher Scientific, Waltham, MA, USA), and the tissue was then dissociated by the trituration with a pipette and vortexed at the maximum speed. Then, the cell suspension was filtered through an 80-μm pore size nylon mesh filter to remove cell clumps, blood vessels, and debris and then the filtrate was centrifuged at 1000*g* for 5 min. The cell pellet was washed with fresh DMEM and filtered through a 40-μm pore size nylon mesh filter. The cells were then suspended in culture DMEM and plated on culture dishes. The cells were cultured in standard conditions (37 °C; 95% air and 5% CO_2_) and the medium was changed twice a week. The FBS concentration in the medium was 20% in the first week, 15% in the second week, and 10% in the third week of culture. In the third week of culture, 0.25 mM dibutyryl cyclic AMP (Sigma-Aldrich, St. Louis, Missouri, USA) was added to the medium to promote the morphological differentiation of astrocytes. After a 3-week incubation period, cultures were approximately 90 to 95% confluent and contained predominantly astrocytes, as shown using antibodies directed against the astrocyte marker glial fibrillary acid protein (GFAP) (Fig. 1a), without contamination of neurons and microglia, as revealed by the absence of NF200 (Fig. 1b) and Iba-1 (Fig. 1c) staining, respectively. The quality of used antibodies was verified by showing positive Iba-1 (Fig. 1d) and NF-200 (Fig. 1e) immunostaining in cultured mouse microglia and neurons, respectively. Positive immunostaining with GluN1 antibody confirmed the presence of GluN1 subunit of NMDA receptor in astrocytes (Fig. 2a). The details of the immunostaining procedure are described in the “Immunocytochemistry” section. All experiments were performed with 3-week-old astrocyte cultures.

### Treatments

After 21 days in culture, the medium was removed and cells were washed twice with phosphate-buffered saline (PBS; 137 mM NaCl, 2.7 mM KCl, 7.3 mM Na_2_HPO_4_, 0.9 mM CaCl_2_, 0.5 mM MgCl_2_, pH 7.4, 37 °C) and exposed to 100 μM NMDA in (i) Ca^2+^ containing medium (DMEM, 10% FBS) for 8 or 72 h, or (ii) Ca^2+^-free medium (DMEM high glucose, no calcium; Gibco, Carlsbad, CA, USA, 10% FBS) for 8 h, or 2 mM Glu in Ca^2+^ containing medium (DMEM, 10% FBS) for 8 h. The incubation time in the Ca^2+^-free medium was limited to 8 h, due to the decreased cell viability at longer incubation times (see the “MTT Assay” section). The DMEMs with and without calcium had the same composition, including the presence of NMDA receptor co-agonist, glycine at 40 mM. NMDA at 100 μM is toxic to neurons (Papadia et al. 2008) but not to astrocytes (Lachmann et al. 2013; Obara-Michlewska et al. 2015) and therefore has been selected to mimic an aspect of slowly progressing astrocytic remodeling occurring in CNS, in diseases encompassing an excitotoxic component.

### Immunocytochemistry

To investigate the purity of the astrocytes culture, the cells were cultured on poly-l-lysine-coated glass coverslips in 24-well plates. The cells seeded onto coverslips were rinsed three times with PBS, fixed with 4% paraformaldehyde/PBS for 20 min at room temperature (RT), washed thrice with PBS, and permeabilized with 0.25% Triton X-100/PBS for 30 min at RT. After three washes with PBS each of 5 min, the cells were blocked in 5% BSA (Sigma-Aldrich, St. Louis, Missouri, USA) or 10% normal goat serum (NGS, Sigma-Aldrich, St. Louis, Missouri, USA) in PBS for 1 h. Then, the cells were immunostained using primary antibodies against GFAP (1:400, mouse monoclonal, MAB360, Merck, Darmstadt, Germany), against NF200 (1:400, N0142, Sigma-Aldrich, St. Louis, Missouri, USA), against Iba-1 (1:500, ab5076, Abcam, Cambridge, UK), and against GluN1 subunit of NMDAR (1:200, 05-432, Merck, Darmstadt, Germany) overnight at 4 °C. After rinsing in PBS, the cells were exposed to goat anti-mouse IgG1 Alexa Fluor 488 or goat anti-mouse IgG (H + L) Alexa Fluor 546, or donkey anti-goat IgG (H + L) Alexa Fluor 546-conjugated secondary antibody (1:500, Invitrogen, Thermo Fisher Scientific, Waltham, MA, USA), for 60 min at RT in the dark. Additionally, the cell nuclei were stained with Hoechst 33258 (H1398, Invitrogen, Thermo Fisher Scientific, Waltham, MA, USA). To obtain the detailed images of the labeled cells, a confocal laser scanning microscope LSM 780 (Zeiss, Jena, Germany) was used. DPSS laser (561 nm) was used to excite Alexa Fluor 546. An argon laser (488 nm) was used for the excitation of Alexa Fluor 488 and diode 405 nm for the excitation of Hoechst. Following the acquisition, the images were processed using the ZEN 2012 (Zeiss, Jena, Germany). These studies were performed in the Laboratory of Advanced Microscopy Techniques, Mossakowski Medical Research Centre, Polish Academy of Sciences.

### MTT Assay

The MTT [3-(4,5-dimethylthiazol-2-yl)-2,5-diphenyl tetrazolium bromide] assay was used as originally described by Mosmann (1983), with slight modifications. The cell cultures were incubated with MTT in DMEM (0.5 mg/mL) for 30 min at 37 °C, thereafter lysed with 96.6% DMSO and the absorbance of the lysate was measured spectrophotometrically at 570 nm. The results were referred to the absorbance of untreated controls, which were taken as 100% value. In incubations in the presence of Ca^2+^, none of the compounds added to the cell cultures affected the outcome of the MTT assay for 72 h, indicating unaltered cell viability under these conditions (Fig. 1f). In the Ca^2+^-free media, however, a slight decrease of cell viability has begun to become apparent when incubations lasted longer than 8 h (data not shown). Therefore, 8-h treatment was chosen for experiments that required a calcium-free medium.

### Silencing of the GluN1 Receptor Gene

Transfection of small interfering RNA (siRNA) into cells has been used for this purpose. Twenty-one nucleotide RNA duplexes specific for the GluN1 subunit of the NMDA receptor (a mixture of 4 specific sequences) were purchased from Qiagen. The cells were transfected according to the manufacturer’s protocol (Qiagen, Hilden, Germany). These cells were treated with NMDA (100 μM; 8 h), 24 h after the onset of transfection, without changing the media. The transfection control (“mock” control) was carried out using HiPerfect reagent. The expression of the GluN1 mRNA in cultures silenced with GluN1 siRNA decreased by 56% and 63% after 8 h and 72 h of transfection, respectively (Fig. 2b).

### Real-time PCR Analysis

The mRNA expression was determined by Taqman Gene Expression Assay (Applied Biosystems), using 1.5 μl of cDNA in a reaction of 10 μl. The assay IDs were Mm00445028_m1 for mouse Kir4.1 (Kcnj10), Mm00802131_m1 for AQP4 (Aqp4), Mm00725701_s1 for GS (Glul), and Mm00607939_s1 for β-actin. The fold change in the gene expression was determined by the 2^-ΔΔCt^ method (Livak and Schmittgen 2001).

### Protein Isolation and Western Blot Analysis

The relative protein contents in cell cultures were determined by Western blotting. The cells were homogenized in radioimmunoprecipitation assay (RIPA) buffer (0.5% CHAPS; Merck, Darmstadt, Germany, 0.01 M sodium phosphate (pH 7.2), 0.15 M sodium chloride, 0.1% sodium dodecyl sulfate, 1% sodium deoxycholate, 1% NP-40; Igepal, Fluka, Bucharest, Romania, 2 mM EDTA). Fifty millimolar sodium fluoride, protease inhibitors (1:200), and phosphatase inhibitors (1:100) (all Sigma-Aldrich, St. Louis, Missouri, USA) were added freshly each time to the RIPA buffer. Protein (20 μg) was separated on 10% SDS-polyacrylamide gel. The blots were incubated overnight with an anti-GS antibody (1:10000; Sigma-Aldrich, St. Louis, Missouri, USA), anti-AQP4 antibody (1:1000; Santa Cruz Biotechnology), or anti-Kir4.1 antibody (1:800; Alomone Labs, Jerusalem, Israel), washed, incubated with HRP-conjugated IgG (1:8000; Sigma-Aldrich, St. Louis, Missouri, USA), and developed using Chemiluminescent Super Signal West Pico Substrate (Pierce, Rockford, IL, USA). After stripping, the blots were incubated with HRP-conjugated anti-GAPDH antibody for 1 h (1:8000; Proteintech, Manchester, UK) and developed as described above. The chemiluminescent signal acquisition and densitometric analysis were conducted by using the G-Box system and GeneTools software (SynGene, Cambridge, UK), respectively. The band intensities (~ 45 kDa for GS, ~ 34 kDa for AQP4, ~ 40 kDa for Kir4.1, and ~ 36 kDa for GAPDH) obtained after densitometry were calculated as GS/GAPDH, AQP4/GAPDH, and Kir4.1/GAPDH ratios and presented in the respective figures as a percent of control.

### Glutamine Synthetase Activity Assay

The specific activity of GS was measured in cell lysates by a colorimetric assay based on the catalysis of γ-glutamylhydroxamate from glutamine and hydroxylamine (Bidmon et al. 2008). GS activity was expressed as micromolar γ-glutamylhydroxamate per minute per milligram of cell protein. The data were presented as percentage (± SD) of the control value.

### Statistical Analysis

**Fig. 3:**
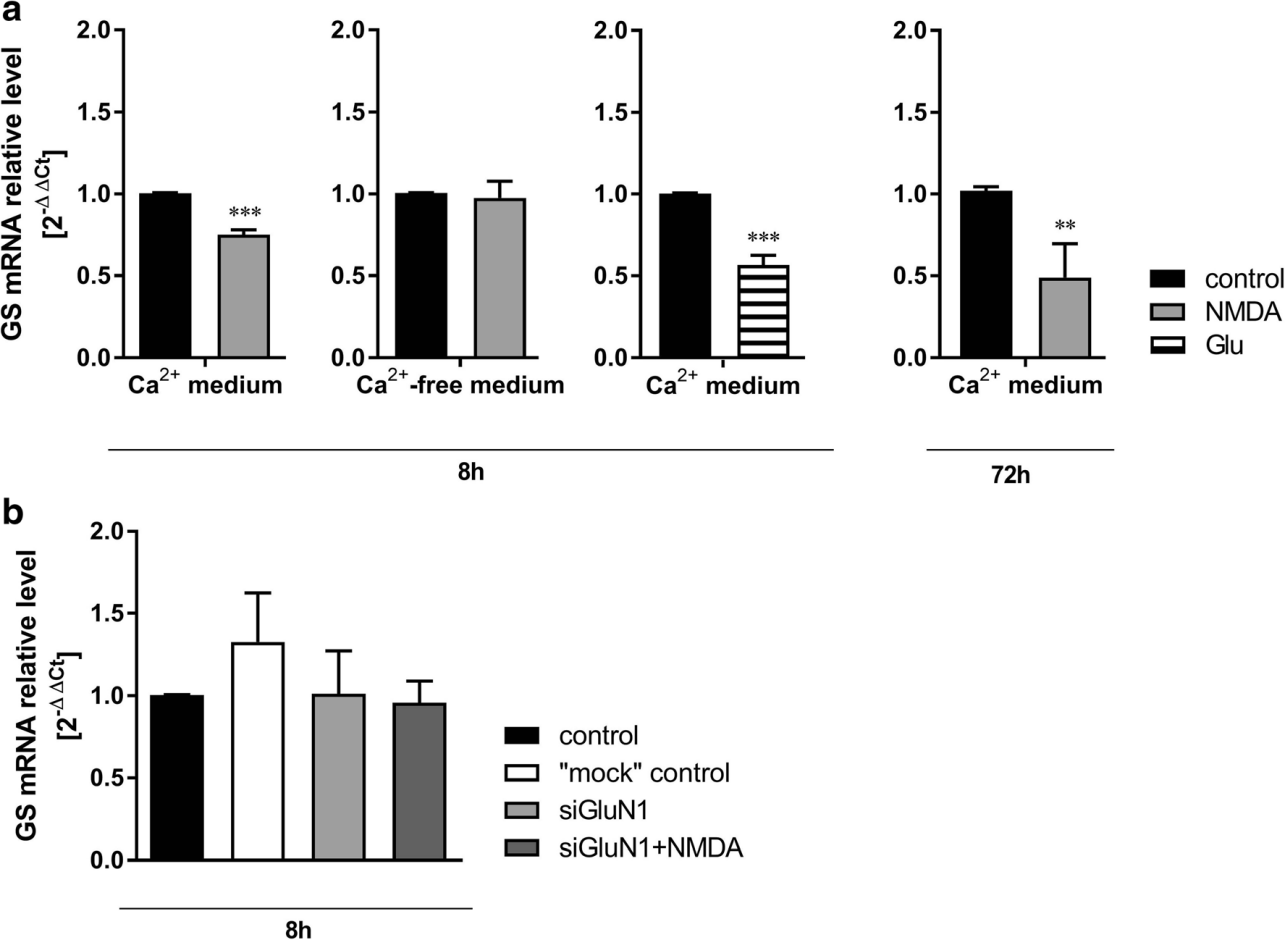
The effect of 8-h or 72-h treatment with NMDA (100 μM) or Glu (2 mM) on glutamine synthetase (GS) mRNA level in mouse cerebral cortical astrocytes cultured in standard or calcium-free medium (**a**) and in standard medium following GluN1 subunit silencing with siRNA (**b**). Data are mean (± SD) from three to four independent experiments. Significant difference vs. control, **P < 0.01; ***P < 0.001 (Student’s *t* test)

**Fig. 4:**
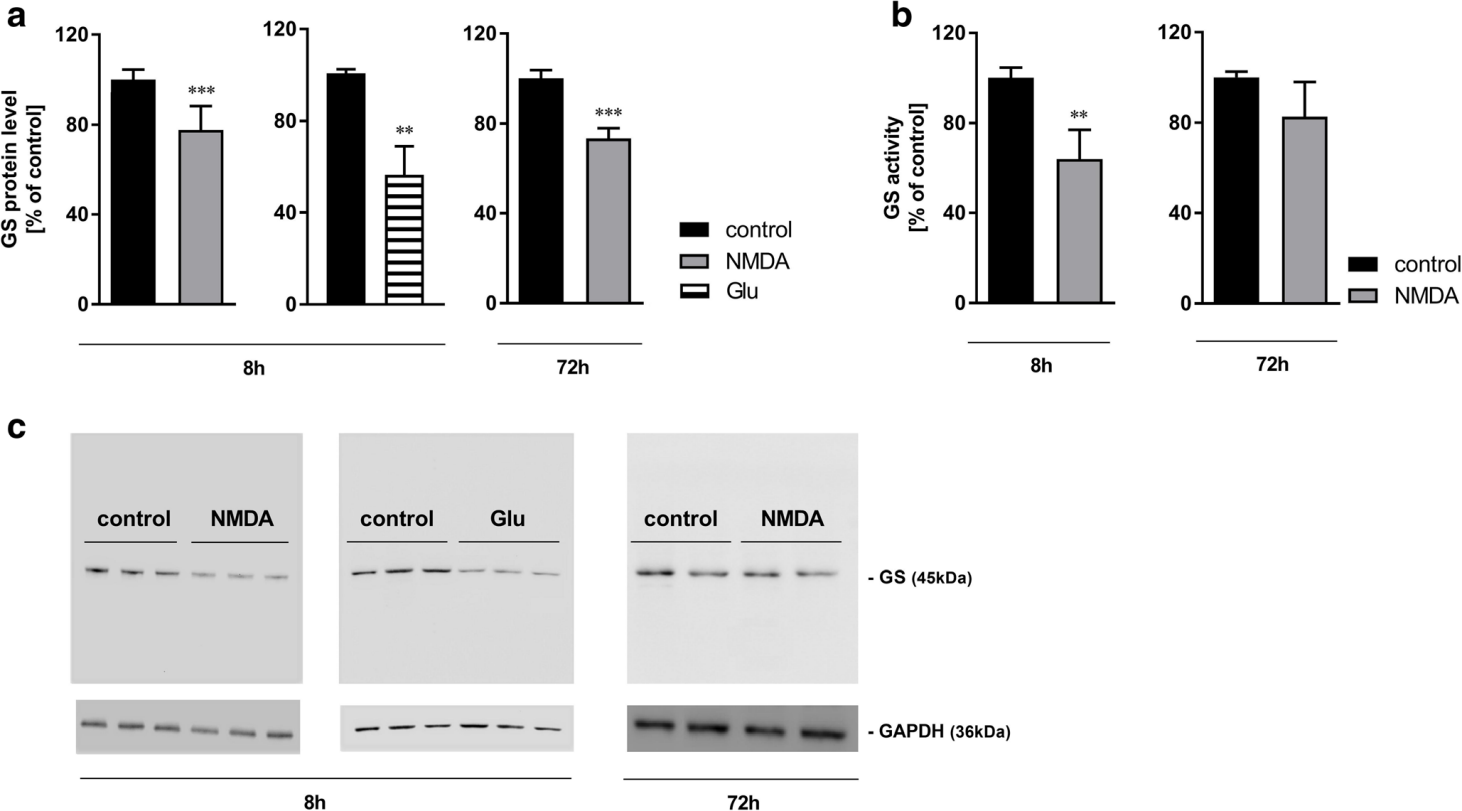
The effect of 8-h or 72-h treatment with NMDA (100 μM) or Glu (2 mM) on GS protein level (**a**) and GS activity (**b**) in mouse cerebral cortical astrocytes cultured in the standard medium. (**c**) Representative Western blot images. Data are mean (± SD) from three to seven independent experiments. Significant difference vs. control, **P < 0.01; ***P < 0.001 (Student’s *t* test)

**Fig. 5:**
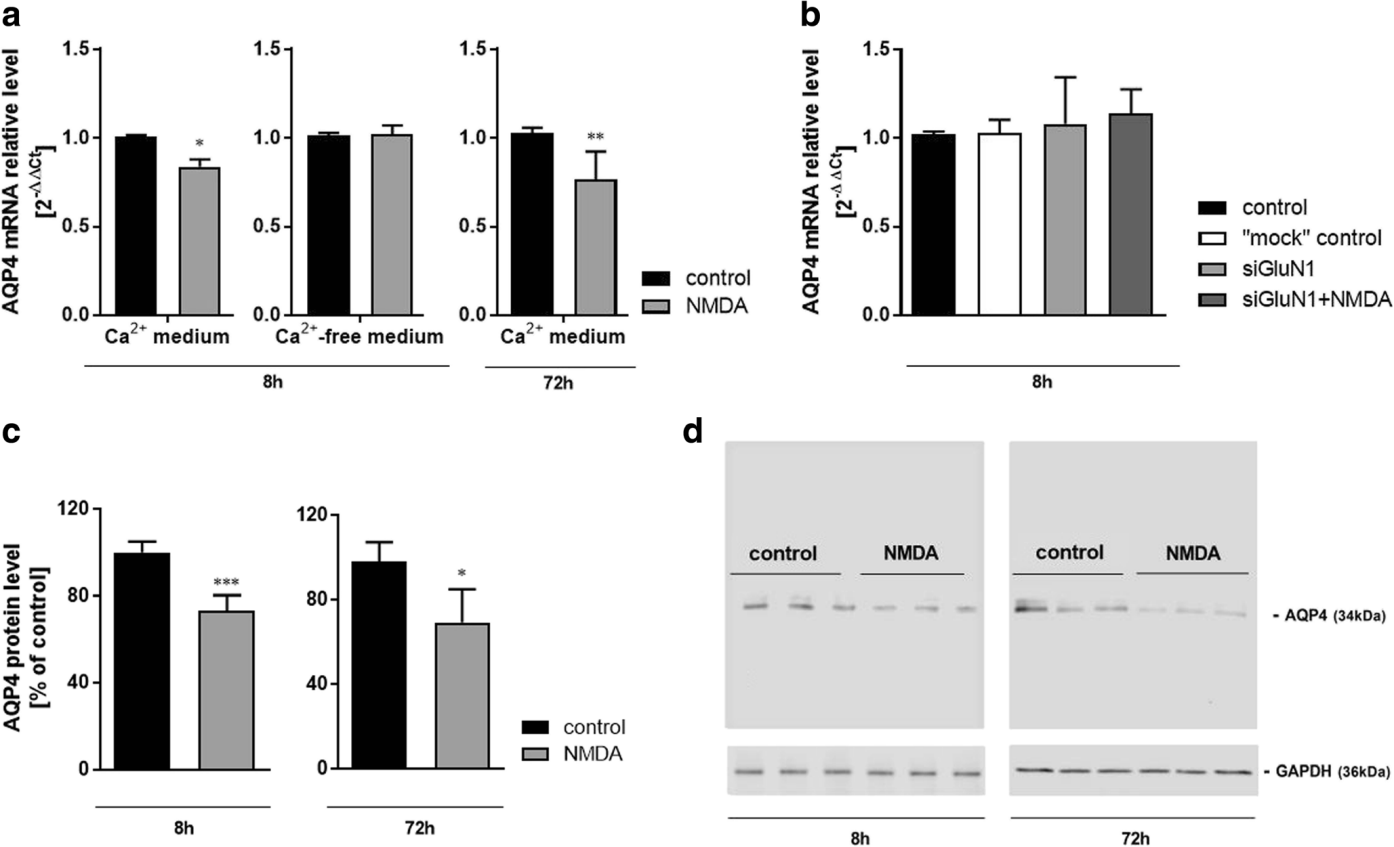
The effect of 8-h or 72-h treatment with NMDA (100 μM) on aquaporin 4 (AQP4) mRNA (**a**, **b**) and protein (**c**) levels in mouse cerebral cortical astrocytes cultured in standard (**a**, **c**) or calcium-free culture medium (**a**) and in standard medium following GluN1 subunit silencing with siRNA (**b**). **d** Representative Western blot images. Data are mean (± SD) from four to five independent experiments. Significant difference vs. control, *P < 0.05; **P < 0.01; ***P < 0.001 (Student’s *t* test)

**Fig. 6:**
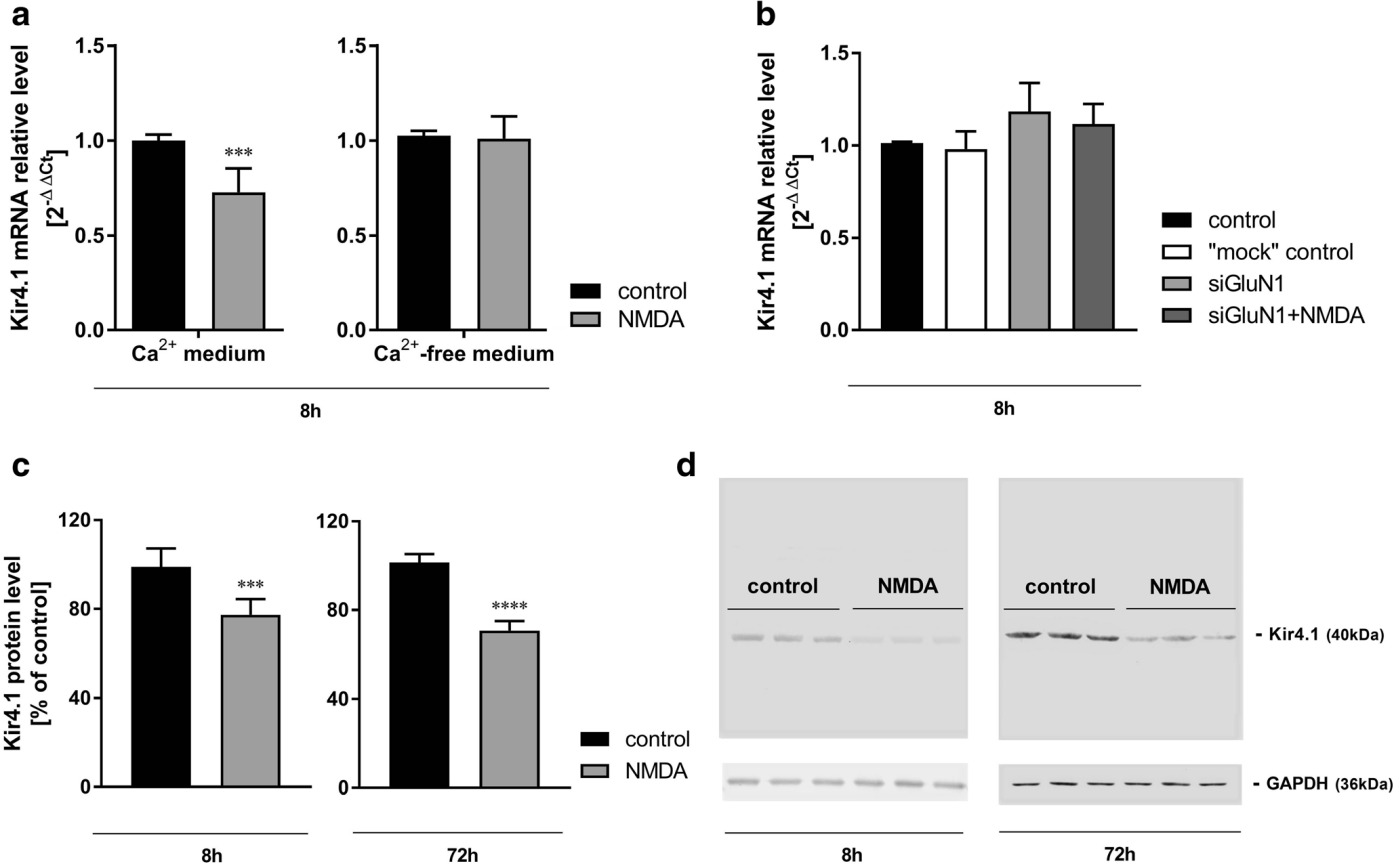
The effect of 8-h or 72-h treatment with NMDA (100 μM) on Kir4.1 mRNA (**a**, **b**) and protein (**c**) levels in mouse cerebral cortical astrocytes cultured in standard (**a**, **c**) or calcium-free culture medium (**a**) and in standard medium following GluN1 subunit silencing with siRNA (**b**). **d** Representative Western blot images. Data are mean (± SD) from  three to  six independent experiments. Significant difference vs. control, ***P < 0.001; ****P < 0.0001 (Student’s *t* test)

The data were analyzed with parametric tests: Student’s *t* test (Figs. 3a, 4a, b, 5a, c, 6a, c) and one-way ANOVA with the Dunnett post hoc test (Figs. 2b, 3b, 5b, 6b). The GraphPad Prism7 software was used to perform the statistical analysis. The Shapiro-Wilk normality test of the real-time data was performed using Statistica software.

## Results

The expression of GS mRNA decreased upon 8-h and 72-h exposure to NMDA (by 25 and 52%, respectively) in the presence but not in the absence of Ca^2+^ ions in the medium (Fig. 3a), and the decrease was abolished in cultures silenced with GluN1 siRNA (Fig. 3b). Eight hours of exposure in the standard medium to Glu (in the presence of Ca^2+^ ions) decreased GS mRNA expression by 44% (Fig. 3a). The GS protein content was decreased by 26% after 8 h (Fig. 4a) and 27% after 72 h of exposure to NMDA (Fig. 4a) in a standard, Ca^2+^-containing, medium. GS protein content was also found to be decreased by 44% in astrocytes exposed in the same medium to 2 mM Glu (Fig. 4a), confirming similarities of responses to NMDA and its physiological agonist previously reported for Kir4.1 expression in rat astrocytes (Obara-Michlewska et al. 2015). The GS activity was decreased by 36% after 8 h and showed a tendency towards a return to the control level after 72 h incubation with NMDA (Fig. 4b).

The expression of AQP4 mRNA decreased upon 8-h and 72-h exposure to NMDA by 17% and 26%, respectively, as compared to the control (Fig. 5a). The decrease at 8 h was noted in the presence but not in the absence of Ca^2+^ ions in the medium (Fig. 5a) and was abolished in cultures silenced with GluN1 siRNA (Fig. 5b). Reduced AQP4 protein contents (26.5% and 22% reduction below control level) were measured at 8 h and 72 h, respectively (Fig. 5c).

Eight hours of exposure to NMDA in standard incubation medium reduced (by 28% as compared to control) the Kir4.1 mRNA expression in astrocytes (Fig. 6a). No change in Kir4.1 mRNA expression was noted in NMDA-treated astrocytes in Ca^2+^-free medium (Fig. 6a), or in cultures in which astrocytes were silenced with GluN1 siRNA (Fig. 6b). The Kir4.1 protein content was decreased by 22% and 29% after 8-h and 72-h exposure to NMDA, respectively (Fig. 6c). The results confirm a previous observation in cultured rat astrocytes, where the reduction of Kir4.1 following incubation with NMDA was noted at both the mRNA and protein levels (Obara-Michlewska et al. 2015).

## Discussion

The main finding of this study is that the sustained exposure of mouse cortical astrocytes to NMDA decreases the expression of three astroglia-specific proteins—GS, AQP4, and Kir4.1 (Figs. 3, 4, 5, and 6). With regard to Kir4.1, the study confirms the effect previously characterized in detail in rat astrocytes (Obara-Michlewska et al. 2015). Abrogation of the NMDA-induced changes by partial knockout of the obligatory NMDAR subunit GluN1 (Figs. 3, 5, and 6) positively verified the specific interaction of NMDA with NMDAR in this case. NMDAR-mediated downregulation of GS, AQP4, and Kir4.1 depended on the presence of calcium in the incubation medium, indicating that the effect was elicited by the canonical, ionotropic mechanism (Figs. 3, 5, and 6). However, in future experiments, the evidence needs to be fine-tuned by assessing the potential effects of the calcium-free medium on intra-astrocytic calcium homeostasis, which appear likely in view of the decreased cell viability at incubations lasting longer than 8 h (data not shown). Of note in this context, the metabotropic or ionotropic mechanisms do not appear to be mutually exclusive, the operation of each depending on the experimental context. For instance, a metabotropic mechanism of NMDAR signaling was observed in astrocytic-neuronal co-cultures durably exposed to low, non-neurotoxic dose of NMDA (20 μM) (Jimenez-Blasco et al. 2015).

Our previous report presented the evidence for NMDAR-dependent decrease of Kir4.1 expression in rats with acute liver failure, a condition associated with excessive extracellular accumulation of Glu (Obara-Michlewska et al. 2015). With regard to GS, the activation of NMDAR in the brain of mice acutely exposed to ammonia decreased the cerebral Gln synthesis (Kosenko et al. 1994). While this result was originally interpreted to ensue tonic activation of neuronal NMDAR, the results of the present study tempt one to consider the contribution of persistently overactivated astrocytic NMDAR. It is tempting to speculate that increased stimulation of NMDAR in astrocytes also contributes to the profound decrease of GS expression/activity in the epileptic foci formed during progression of the temporal lobe epilepsy (TLE), a process associated with periodic increases of the excitatory neurotransmission (Eid et al. 2008; van der Hel et al. 2014; Swamy et al. 2011). A similar mechanism may contribute to the loss of GS recorded in autopsy material of Alzheimer’s disease (AD) patients (Robinson 2000) and astrocytes derived from animals with experimentally modeled AD (Olabarria et al. 2011; Kulijewicz-Nawrot et al. 2013). However, in other CNS pathologies associated with excitotoxicity, the direction and magnitude of the responses of GS vary from the clinical setting to model and from the intensity and duration of the excitotoxic insult (for a recent review, see Jayakumar and Norenberg 2016). In turn, the loss of AQP4 may cause ion and water imbalance, among other mechanisms by impairing the clearance of interstitial solutes via the glymphatic system, a situation modeled in Aqp4-null mouse brains (Iliff et al. 2012). However, similar to GS, AQP4 expression varied in different experimental models of epilepsy (Eid et al. 2005; Lee et al. 2004; Lee et al. 2012), ischemic/anoxic insult (Aoki et al. 2003; Hoshi et al. 2011), or hepatic encephalopathy (Rama Rao et al. 2010; Wright et al. 2010; for reviews, see Badaut et al. 2014; Saadoun and Papadopoulos 2010; Potokar et al. 2016). Therefore, the causal relation between the exposure of astrocytic NMDAR to excess Glu and the GS or AQP4 status will have to be studied in vivo for each individual pathological condition.

In conclusion, the present study demonstrates that prolonged overexposure of astrocytes in vitro to NMDA downregulates the expression of three astrocytic proteins critical for regulation of ion (Kir4.1), water (AQP4), and neurotransmitter balance (GS), respectively. As such, the study discloses some novel aspects of astroglial plasticity driven by NMDAR in response to the excitotoxic signal. Depending on the nature and the duration of action of the pathogenic factor, NMDAR-induced changes in astrocytic function may either be neuroprotective or detrimental to the brain. In the present experimental setting, downregulation of GS was translated to the decreased enzyme activity. In the future, studies in more complex systems will have to be carried out to assess whether and in what degree the NMDAR-mediated loss of Kir4.1 and AQP4 bears upon the specific functions of the proteins.
